# Episodic Memory in Detoxified Alcoholics: Contribution of Grey Matter Microstructure Alteration

**DOI:** 10.1371/journal.pone.0006786

**Published:** 2009-08-26

**Authors:** Sandra Chanraud, Claire Leroy, Catherine Martelli, Nikoleta Kostogianni, Françoise Delain, Henri-Jean Aubin, Michel Reynaud, Jean-Luc Martinot

**Affiliations:** 1 Inserm, U797 Research Unit “Neuroimaging & Psychiatry”, IFR49, Orsay, France; 2 CEA, “Neuroimaging & Psychiatry” U797 Unit, Hospital Department Frédéric Joliot & Neurospin, I2BM, Orsay, France; 3 Univ Paris Sud, UMR U797, Orsay and Univ Paris Descartes, UMR U797, Paris, France; 4 Department of Psychiatry and Behavioral Sciences, Stanford University School of Medicine, Stanford, California, United States of America; 5 Neuroscience Program, SRI International, Menlo Park, California, United States of America; 6 APHP, Department of psychiatry and addictology, Paul Brousse Hospital, Villejuif, France; 7 APHP, Addiction Treatment Center, Emile Roux Hospital, Limeil-Brevannes, France; National Institute on Drug Abuse, National Institutes of Health, United States of America

## Abstract

Even though uncomplicated alcoholics may likely have episodic memory deficits, discrepancies exist regarding to the integrity of brain regions that underlie this function in healthy subjects. Possible relationships between episodic memory and 1) brain microstructure assessed by magnetic resonance diffusion tensor imaging (DTI), 2) brain volumes assessed by voxel-based morphometry (VBM) were investigated in uncomplicated, detoxified alcoholics.

Diffusion and morphometric analyses were performed in 24 alcohol dependent men without neurological or somatic complications and in 24 healthy men. The mean apparent coefficient of diffusion (ADC) and grey matter volumes were measured in the whole brain. Episodic memory performance was assessed using a French version of the Free and Cued Selective Reminding Test (FCSRT). Correlation analyses between verbal episodic memory, brain microstructure, and brain volumes were carried out using SPM2 software.

In those with alcohol dependence, higher ADC was detected mainly in frontal, temporal and parahippocampal regions, and in the cerebellum. In alcoholics, regions with higher ADC typically also had lower grey matter volume. Low verbal episodic memory performance in alcoholism was associated with higher mean ADC in parahippocampal areas, in frontal cortex and in the left temporal cortex; no correlation was found between regional volumes and episodic memory scores. Regression analyses for the control group were not significant.

These findings support the hypothesis that regional microstructural but no macrostructural alteration of the brain might be responsible, at least in part, for episodic memory deficits in alcohol dependence.

## Introduction

Episodic memory is a neurocognitive system that enables conscious recollection of personal life events from one's past and mental projection of anticipated events into one's subjective future [1]. Episodic memories require encoding, storage and retrieval of personally experiences events. It is widely known that alcoholism may cause deficits in episodic memory. More specifically, impairment in episodic memory in alcoholism may be associated with reduction in the ability to learn complex novel information [2].

Such dysfunction makes behavioral change difficult for heavy drinkers and thus may hamper successful therapeutic intervention. Whereas studies of learning abilities have provided some initial information regarding effects of alcoholism on episodic memory, the specific nature of this impairment (for example encoding vs retrieval processes) has never been clearly determined. Some studies have shown retrieval deficits in alcoholic patients [3], [4], notably in tasks involving executive control, such as free recall [3], while the preservation of these retrieval processes has also been reported [5]. As a result, the examination of brain regions implicating episodic memory impairment in alcohol dependence could further the understanding of this neurocognitive system alteration in alcoholics.

Even though uncomplicated alcoholics may likely have episodic memory deficits, discrepancies exist regarding to the integrity of brain regions that underlie this function in healthy subjects. Atrophy of the medial temporal lobe, particularly the parahippocampal and hippocampal regions, has been associated with memory loss in several diseases such as in early Alzheimer's disease [6]. However, a meta-analysis of 33 studies examining relationship between hippocampal volume and memory performance yielded an extreme variability and surprisingly little support for the “bigger-is-better hypothesis” in elderly adults [7]. Thus, the relationship between hippocampal *volume* and memory also remains unclear.

For instance studies of hippocampal alteration in alcoholism have produced discrepant findings depending on the techniques used. Magnetic resonance imaging techniques revealed volume reductions in medial temporal structures (i.e. the amygdala, hippocampus and parahippocampal gyrus, the insula and most adjacent cortices) [8], [9]. Conversely, neuronal counting techniques have revealed no neuronal loss in alcohol dependent subjects [10]. Other studies have reported glial cell loss [11] and neuronal dysmorphology [12] rather than neuronal loss in this region. Hippocampal volume shrinkage has been attributed also to pathological changes in white matter (decrease in axonal diameter and loss of white matter) [12], and the incorporation of newly formed neurons to the dentate gyrus could also be affected by alcohol [13], [14]. Furthermore, Sullivan et al. [15] suggested that a moderate hippocampal volume loss may not be adequate to cause significant memory impairment on its own.

Multiple regions of the brain may be involved in memory-related operations: frontostriatal and medial-temporal circuits are viewed as especially important in encoding, maintenance, and retrieval of information [16], [17], [18], [19], [20]. Therefore, alterations in nodes or connections within circuits, such as alteration in sub-cortical and cortical structures connected to the hippocampus via the Polysynaptic Pathway [21], could also be involved in episodic memory impairment in uncomplicated alcoholism.

However, microscopic alterations such as dendritic and synaptic changes that have been documented in uncomplicated alcoholics [22] might, more likely, underlie episodic memory dysfunction. Indeed, these changes might be precursors of more severe structural changes and might be, at least in part, responsible for the functional changes and cognitive deficits revealed early in alcohol dependence [10].

Since the relationships between episodic memory and regional volumes or microstructure remain unclear, we assessed the effect of regional microstructural and volume alteration on episodic memory processes in patients with alcohol dependence. We hypothesized that microstructural changes in temporal, parahippocampal and hippocampal areas, more than volumetric changes in these regions or elsewhere in the brain, could be related to episodic memory impairment.

Diffusion tensor imaging (DTI) provides a unique insight into the cellular integrity of the brain. Moreover, diffusion measurements have been shown to be better correlated with cognition than conventional MRI approaches [23]. The Apparent Coefficient of Diffusion (ADC), which is a measure of the rate of microscopic water diffusion thus providing an estimate of water motility in a voxel [24], can be used to determine microstructure changes within a brain region.

Many studies have shown brain defects in relation to neurocognitive dysfunction in abstinent alcoholics, but few have related MRI volumetric measures, DTI, and memory assessment at the same time. We compared ADCs using DTI, and grey matter volumes using Voxel-Based-Morphometry (VBM) methods, to assess regional whole-brain changes between alcoholics and healthy controls. Then, we examined relationships between microstructure integrity, regional volumes and verbal episodic memory performance in alcoholic subjects. We hypothesized that the ADC values in temporal regions would account for the verbal memory performance in alcoholics.

## Materials and Methods

### Participants

Participants were 24 patients who met DSM-IV criteria for alcohol dependence and 24 healthy participants. All were Caucasian, right-handed men. Patients were recruited upon admission to detoxification or day-care units in the addiction department of the Paul Brousse Hospital in the Paris area (Assistance Publique, Hôpitaux de Paris). Senior psychiatrists (JLM and CM) interviewed and clinically evaluated patients, as well as examined their medical records and biological data. Medical consequences of chronic alcoholism on other organs (e.g. alcoholic cirrhosis) may impact both brain microstructure and behavioral performance [25]. Thus, we selected detoxified drinkers with moderate alcoholism, *i.e.* with no clinical evidence of brain dysfunction or medical conditions considered to be clinical indicators of severe alcoholism (e.g. alcohol-induced dementia or chronic liver disease [25]). The inclusion criteria, at time of the study, were: 1) fewer than three periods of withdrawal; more than two periods of withdrawal may be associated with greater cognitive impairment [26], 2) detoxification treatment for at least three weeks, and abstinence as assessed by levels of gamma-glutamyl-transferase and carbohydrate-deficient transferrin in normal range (see Table 1), and 3) no use of sedative medications for at least seven days preceding the study (before participating in this study, the patients had been treated during withdrawal with decreasing doses of lorazepam and vitamins B1 and B6). The exclusion criteria included: 1) signs or symptoms of malnutrition, 2) signs of liver dysfunction: aspartate aminotransferase/alanine aminotransferase ratio greater than two [27], and 3) a score>20 on the Hamilton Anxiety Scale or a score>10 on the Hamilton Depression Scale.

**Table 1:** Means and standard deviations (±SD) of demographic variables for the alcohol-dependent (n = 24) and healthy (n = 24) subjects, and biological variables for alcohol-dependent subjects.

	Alcoholics	Controls	*p* value^*^
Age (years)	47.8±7.7	45±5.6	0.18
BMI (kg/m^2^)	24.2±3.88	24.7±3.4	0.39
Years of education	7.75±2.99	8.7±3.37	0.17
AUDIT	32.7±4.21	2±1.8	0.00
MMSE	28.9±2.2	29.2±1.2	0.12
SAS score	1.65±0.37	1.53±0.16	0.12
**Biological variables**			
GGT	49.2±60.4 (<53) ^a^		
ALT level (U/liter)	25.95±13.76 (<38)^ a^		
AST level (U/liter)	27.33±13.78 (<40)^ a^		
AST/ALT	1.08±0.23		
CDT	1.97±0.25 (<2.6)^ a^		

BMI: body mass index; AUDIT (range 0–40): Alcohol Use Disorders Identification Test; MMSE: Mini-Mental State Examination; SAS: Social Adjustment Scale. ^a^Laboratory norms. AST: aspartate aminotransferase; ALT: alanin aminotransferase; GGT: gamma-glutamyl-transferase; CDT: carbohydrate-deficient transferring.

We recruited healthy control participants from the community. Inclusion criteria were: 1) an alcohol consumption less than two equivalent standard alcoholic drinks per week and 2) a score less than or equal to five on the Alcohol Use Disorders Identification Test (AUDIT; [28]).

For both groups, exclusion criteria comprised being under 25 or over 65 years of age, being left-handed, non-fluent in French, drug abuse (other than nicotine), anxiety or depressive disorders and neurological, somatic or other psychiatric symptoms, having a history of head injury requiring hospitalization or the need for anticonvulsants or other medication that may compromise brain functioning, contraindications to MR examination (pregnancy, indwelling metallic hardware, joint replacement, etc.), stroke, or other major brain abnormalities observed on MRI scans.

The study was approved by the Bicêtre ethics committee. After receiving an explanation of study procedures and aims and questions were answered, participants provided written informed consent to participate.

### Memory assessment

Participants received the Mini Mental State Examination (MMSE; [29]) and a neuropsychological assessment on the day of or up to three days after the MRI examination. Also, participants were assessed on the AUDIT scale and the Social Adjustment Scale Self Report [30]. The SAS-SR is a self-report questionnaire that evaluates daily functioning, and includes questions on social and leisure activities, relationships with the marital partner, children and extended family, and perception of economic status.

We selected a French version of the Free and Cued Selective Reminding Test verbal episodic memory assessment [31]. During this task, in order to control for encoding, the participant had to identify each of the 16 words to be remembered by pointing and reading aloud in response to its semantic category. All 16 words have to be retrieved at immediate cued recall before memory assessment begins to ensure the participants have encoded all the items. Recall is first assessed through free recall, then through cued recall for the missing words. This procedure is repeated three times to give the subject the opportunity to improve performance and provides two main scores: free and total (free+cued) recall. The performance in both free and total recall was scored by number of recalled words in the three sessions. In order to evaluate the specificity of brain correlates of verbal episodic memory in alcoholics, we also investigated recall of semantic information (WAIS III Information subtest; [32]) which is believed to be processed and stored at a cortical level; and then examined the relationship between subjects' performance and whole-brain imaging data (microstructure and volumes).

### Brain imaging

#### Anatomical image acquisition

All participants underwent volumetric MRI brain scanning using a 1.5T imager (General Electric Healthcare, Milwaukee, WI) with a standard 3D T1-weighted inversion recovery fast-spoiled gradient-recalled (IR-FSPGR) sequence with the following parameters: axial orientation, matrix = 256×192 interpolated to 256×256, 124 slice locations, 0.9375×0.9375 mm^2^ in-plane resolution, slice thickness = 1.3 mm, TE = 2 ms, TR = 10 ms, TI = 600 ms, flip angle = 10°, read bandwidth = 12.5 kHz.

#### Image preprocessing of anatomical data

All MRI data were processed using SPM2 software (Wellcome Department of Cognitive Neurology, London; (http://www.fil.ion.ucl.ac.uk/spm)) running on MATLAB version 7 (The MathWorks, Natick, MA).*Voxelwise statistics of anatomical data*. MRI images were analyzed using the optimized approach of VBM developed by Good *et al*., [33]. This is a fully automated whole-brain technique that provides a voxel-wise assessment of regional cerebral matter.

#### DTI acquisition

Diffusion-weighted images were acquired with an echo-planar imaging (EPI) sequence (TE = 70 ms, TR = 8300 ms, b-value = 700 s/mm^2^, 56 slices of 2.4 mm, axial orientation, in plane resolution = 0.9375×0.9375 mm, matrix 128×128). The DTI acquisition included five supplementary T_2_ images without diffusion weighted (b = 0) and 36 images with diffusion gradients (b = 700 s/mm^2^) applied along 36 non-collinear directions. The acquisition process was designed to allow reconstruction of the diffusion tensor, even if subjects had moved during the scanning procedure [34]. The number of orientations used to reconstruct the diffusion tensor was similar in the patient and control groups (34.6±2.7 and 35.3±1.9, respectively; t = −1.053, p = 0.3).

#### DTI images preprocessing

Processing of the DTI images was performed using Brainvisa 3 software (www.brainvisa.info/) and included (1) the separation of the images without diffusion weighting (b = 0) from diffusion-weighted ones, (2) the correction of diffusion images for echoplanar distortion, (3) the evaluation of the diffusion tensor on the basis of the corrected diffusion images and (4) the voxel-wise calculation of the ADC on the basis of the diffusion tensor.*Voxelwise statistics of DTI data*. The individual maps of ADC were submitted to analysis tools for voxel-based analyses, using SPM2. SPM2 analysis was conducted according to the one used by Burns [35] in schizophrenic patients. Due to slight differences in the applied methods, the single steps of the voxel-based analysis used in the present study were as follows: an appropriate diffusion template was created by applying to native diffusion images, non-linear transformations determined by normalizing each T2-weighted (b = 0) EPI image onto the MNI EPI template. Native ADC maps were then normalized using this template, resampled at an isotropic voxel size of 2 mm^3^ that represents a voxel size similar to the originally acquired voxel size, and smoothed with an 8 mm-FWHM (full width at half maximum) isotropic Gaussian function.

### Statistical analyses

We tested the normality of the distribution of neuropsychological data using SPSS 8.0 software (SPSS Inc., Chicago, Ill.) and then converted the raw data to *z* scores by adjusting for age and level of education. Statistical significance of the between-group differences for demographic data was calculated by using Student's t-test methodology (Table 1).

#### Between-group analysis

Between-group comparisons of the ADC and of the grey matter volumes were carried out on a voxel basis using the general linear model, based on random Gaussian field theory [36]. Age was used as a covariate in the ANCOVA of both voxel-based volumes and ADC values. The height threshold for comparison analyses was set at *p*<1.10^−3^ FDR-corrected (false discovery rate) with a spatial extent of 100 voxels. Significant peak voxels were reported in MNI (Montreal Neurological Institute) template coordinates. Their regional location was obtained by overlaying the *t*-maps on the study-specific template and using the AAL [37] atlas accompanying the MRIcro software package [38].

#### Regression analyses

We hypothesized that participants with alcoholism would have lower scores in free recall trials as alcoholics have been shown to exhibit deficits in retrieval [3], [4]. Thus, we explored the relationships between brain measures and free recall Z-scores by regression analyses of the whole-brain imaging data in both groups separately. Regression analyses were carried out for 22 alcoholics because 2 patients did not complete the neuropsychological assessment.

SPM t-maps from the between-group comparisons were used as a mask [39]. Thus, linear regression analyses were carried out in each voxel that had been highlighted by the between-group analyses. In addition, multiple regression analyses between clinical measures (e.g. duration of alcohol dependence, length of total drinking history, etc.) and grey matter volume and ADC respectively were carried out within the alcohol dependent group. All regression analyses were carried out with an exploratory uncorrected statistical threshold set at *p*<0.001 with a minimal cluster size of 10 voxels.

## Results

In alcoholics, the mean age of first drink was in early adulthood (Mean: 20.96±Standard Deviation: 6.38), whereas the reported mean age of onset of dependence was 39.75±10.33. During the 3 months preceding the current detoxification, patients drank a mean of 24.4±14.35 drinks (10 g of ethanol) per day, with a mean of duration of dependence of 9.2±8.9 years. The time of abstinence at testing time was from 3 weeks to 2 years with a mean of 31±31 weeks. Most were active tobacco smokers. The groups were similar in mean age, body mass index (BMI), MMSE scores, and social functioning (Table 1).

The between-group comparison of verbal memory performance indicated significant effect of group on free recall (sum Z-scores: Alcoholics: −1.77±3.07; Controls: 1.68±2.65; t (44) = 4.34; *p*<0.001) but no significant effect on the total recall measures in the FCSRT. A memory score corresponding to the sum of the three free recall Z-scores [40] was used for the regression analysis. Also, the between-group comparison of semantic performance indicated significant effect of group on WAIS-information scores (sum Z-Scores: Alcoholics: −1.7±1.39; Controls: 0.03±1.37; t (44) = −4.23; p<0.001).

**Figure 1:**
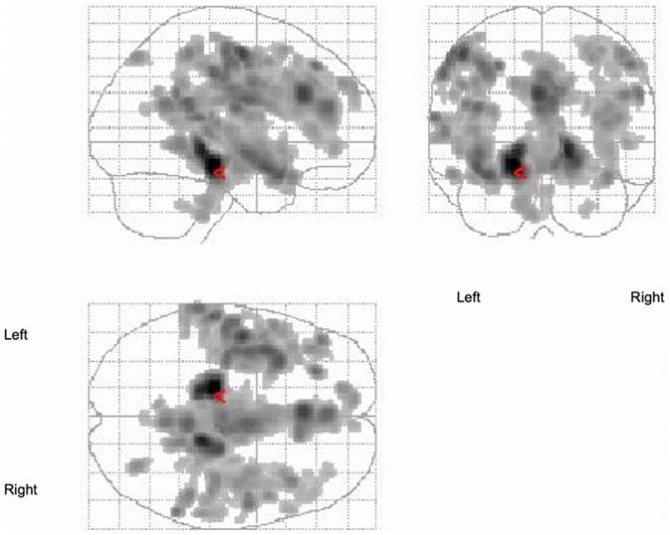
Between groups comparison of ADC images. p<0.001 FDR corrected.

Between-group comparisons of ADC showed significant clusters with increased ADC in alcoholics mainly in temporal and frontal regions (See Figure 1) and in the cerebellum (Table 2). No region with increased ADC was revealed in the control group when compared to alcoholic group.

**Table 2:** Regions of significant increases in ADC in alcoholics compared to healthy subjects.

Regional clusters	Side	Cluster size: voxel number^*^	MNI coordinates of peak voxel			Maximal Z-value
Parahippocampal gyri and hippocampi	L,R	21 441	−16	−26	−14	6.23
Pre- and postcentral gyri	L	2308	−48	−8	52	5.56
Superior temporal gyrus	L	5563	−33	14	−20	5.36
Frontal gyrus	L	740	−27	34	46	5.08
Pre- and postcentral gyri	R	560	52	−4	46	5.03
Supramarginal gyrus	R	271	57	−48	30	5.01
Inferior parietal gyrus	R	485	48	−40	52	4.85
Precentral gyrus	L	327	−48	4	34	4.77
Superior temporal	L	118	−54	10	−18	4.71
Superior parietal	R	155	30	−72	51	4.69
Middle frontal gyrus	R	198	34	16	56	4.56
Middle frontal gyrus	R	224	58	−32	46	4.53
Superior temporal	R	107	50	−30	0	4.46
Inferior frontal gyrus	R	115	56	20	27	4.27
Middle frontal gyrus	R	481	44	27	42	4.20
Cerebellum	R	108	26	−36	−42	4.11

R = Right, L = left. Regional clusters of voxels are presented by decreasing Z value. ^*^voxel size: x = 2, y = 2, z = 2 mm. MNI: Montreal Neurological Institute.

**Figure 2:**
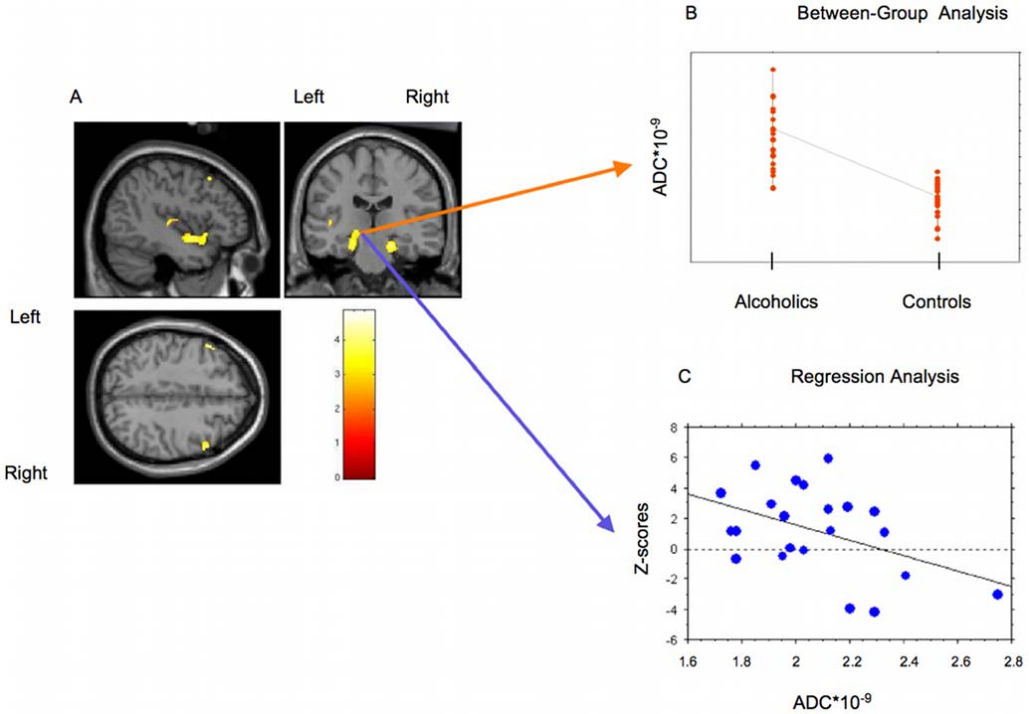
Between groups and regression analyses in the left parahippocampus gyrus. A/Regions where negative correlation between free recall scores and the ADC was detected in alcoholics are rendered on a template image. The colored bar represents the T-score of significant voxels. B/Mean ADC in the left para-hippocampus gyrus (MNI coordinates: x = −16,y = −26,z = −8) of alcoholic and healthy control subjects. C/Plotted values of verbal memory Z-scores and mean ADC cluster values, in alcoholdependent group at the highest peak-voxel (left para-hippocampus gyrus) detected by the regression analysis (MNI coordinates: x = −16,y = −26,z = −8; R^2^ = 0.184; p = 0.046).

Regional regression analysis between memory performance and ADC in alcoholics (Figure 2A) revealed negative correlations bilaterally in the hippocampus and parahippocampus gyri (x = 20,y = −20,z = −20, 770 voxels, Z = 3.90; x = −16,y = −26,z = −8, 498 voxels, Z = 3.32), bilaterally in the middle frontal gyri (x = −52,y = 21,z = 38, 255 voxels, Z = 3.84; x = 52,y = 14,z = 44, 183 voxels, Z = 3.37) and in the left superior temporal gyri (x = −44,y = 8,z = −12, 451 voxels, Z = 3.38; x = −45,y = −15,z = 8, 158 voxels, Z = 2.98).

The semantic performance (WAIS-information) was correlated with ADC values in left (x = −44; y = 27; z = −16) and right (x = 30; y = 33; z = 0) frontal regions.

Whereas smaller volumes in alcoholics were revealed in brain regions where ADC was higher in these participants, no significant correlation was found between brain region volumes and free recall scores in alcohol dependent group (Table 3).

**Table 3:** Regions of significant volume decreases in alcoholics compared to healthy subjects.

Regional clusters	Side	Cluster size: voxel number^*^	MNI coordinates of peak voxel			Maximal Z-value
Hippocampus	R	530	15	−32	2	Inf
Postcentral gyrus	L	1684	−45	−6	57	7.55
Postcentral gyrus	R	376	52	−3	51	7.49
Posterior cingulum	R,L	4432	3	−36	30	7.03
Middle frontal gyrus	L	580	−40	39	33	6.94
Supramarginal gyrus	R	116	58	−32	50	6.92
Inferior parietal gyrus	R	224	48	−40	52	6.78
Hippocampus	L	267	−15	−33	0	6.72
Precentral gyrus	L	211	−52	8	39	6.29
Middle frontal gyrus	R	254	51	30	32	6.27
Inferior frontal gyrus	R	115	57	22	24	6.22
Inferior parietal	R	341	52	−40	52	6.06
Superior temporal gyrus	L	258	−66	−24	4	5.89
Middle frontal gyrus	L	148	−8	57	33	4.57

R = Right, L = left. Regional clusters of voxels are presented by decreasing Z value. ^*^voxel size: x = 2, y = 2, z = 2 mm. MNI: Montreal Neurological Institute.

On the contrary, as well as ADC values, grey matter volumes in frontal regions were correlated with alcoholics' WAIS information Z-scores.

None of the regression analyses carried out between these data was significant for the control group.

Finally, none of the regression between alcoholism-related measures (e.g. duration of dependence, age at onset of dependence, duration of abstinence) and grey matter volume and ADC respectively was significant in alcohol dependent group.

## Discussion

The relationships of episodic memory performance to ADC and to grey matter regional volume were investigated in patients with alcohol dependence. Alcoholics were impaired in the free recall task but not in the total recall one, thus confirming the findings of most previous studies reporting an impairment of episodic retrieval abilities [2], [41], [42], [43]. Main results showed that alcoholics had widespread increase of ADC in multiple brain regions. Higher ADC was found mainly in frontal and temporal regions and in the cerebellum. Also, alcoholics had lower grey matter volumes in all of these regions. However, significant relationships were revealed only between episodic memory scores and ADC (not with volumes) in these regions.

Molecular diffusion results from a random, microscopic translational motion of water molecules and the water mobility as restricted by cell membranes (barriers). The changes in ADC and thus in water mobility are consistent with alterations within brain regions and are reflective of tissue remodeling that may include changes in microglia, astrocytes and axons within the affected region. Alcohol-related brain changes may be related to cell body death, and/or loss of axons, dendrites, and synapses, however it is difficult to determine the sequence of events and the causal relationships among these events.

In alcoholics, regions with higher ADC typically also had lower grey matter volume. These findings, in conjunction with the significant correlation between ADC measures and episodic memory scores on one hand and the non-significant correlation between grey matter volumes and episodic memory scores on the other hand, lead us to speculate that microstructural abnormalities are related to, and could be, the primary step of the neuronal degradation process and subsequent atrophy [44]. Further longitudinal studies, however, are necessary to disentangle putative relationships between microstructure, regional volumetry, degeneration and regeneration processes.

We have found clear links between higher ADC in frontal, temporal, hippocampal and parahippocampal regions and lower verbal episodic memory performance, which suggest that higher microscopic water diffusion in these brain regions is associated with degraded memory performance. Present findings support the theory that microstructural changes in the brain in alcoholics may result in cognitive impairment as previously shown in aging [45] or in patients with mild cognitive impairment [46]. The location of the significant correlations coincided with neural areas known to be highly involved in this kind of task (e.g. the hippocampus and the parahippocampal, frontal and temporal cortex). The greatest correlation found with left regions in our study may reflect the verbal nature of the task [47]. Moreover, regarding the medial temporal regions, previous fMRI studies showed activations in the parahippocampal region when healthy subjects are engaged in verbal episodic memory processes. Indeed, the entorhinal cortex activity during word encoding correlated with subsequent test performance and the peri-rhinal cortex was activated during the episodic intra-item encoding [48]. Present findings are also concordant with another functional imaging study that revealed that successful encoding into episodic memory engages neural circuits in the posterior part of the hippocampus [49]. The hippocampus has been linked to binding processes as well as to conscious recollection of recent events [19], [50]. Because regional alterations in brain microstructure were detected in the present alcoholic sample in most regions known to support verbal episodic memory (medial temporal lobe), these findings add further evidence for *genuine* episodic memory deficits in alcoholism [2].

Few recent studies have investigated hippocampo-neocortical networks and their contribution to episodic memory. Ranganath et al. [51] identified several frontal and temporal cortical regions that showed enhanced coupling with the left hippocampal region during the processing of objects that were subsequently remembered relative to those subsequently forgotten, suggesting a heightened correlation of hippocampo-neocortical interactions during successful memory formation. Alterations within the system consisting of the hippocampus and its connections to the mammillary bodies and to the anterior thalamic nuclei, presumed to mediate recollection relying on relational information, may cause deficits in memory for relational information that typifies contextual memory, while cued recall is spared. Consistently, impaired free recall and preserved cued recall were observed in the alcoholics studied here.

It is widely agreed that the prefrontal cortex plays a key role in controlling episodic retrieval in general and resolving interference in particular (for a review, see [52]). More specifically, it was shown that damage to the frontal lobes could lead to increase interference susceptibility, probably due to difficulties in inhibiting irrelevant memory contents [53]. Then, as suggested by the HERA model [54], correlations found bilaterally in prefrontal cortex may reflect that alcoholic subjects have difficulties both in encoding and in retrieving information. Accordingly, ‘effortful’ free recall of words (during the Free and Cued Selective Reminding Test) was correlated with right frontal metabolism in patients with Alzheimer's disease [55]. These findings are in keeping with the important interactions between the temporal structures and the frontal lobe during encoding and retrieval in episodic memory [56]. Neural models of long-term memory often describe retrieval as an interaction between posterior “storage systems” and an anterior “control system”. In effect, executive functions are known to contribute to the strategic organization of information in order to facilitate encoding and retrieval in memory [57]. Also, executive functions permit to maintain items in a fixed sequence and integrate diverse types of information (factual, temporal, spatial) into a meaningful representation [1] in order to form a comprehensive episode [58]. Thus, frontal alterations could contribute to episodic memory impairment even if alcoholic patients present genuine episodic memory deficits that cannot be regarded solely as the consequences of executive dysfunctions [2].

Notably, even though the results found in the cerebellum are in agreement with previous studies showing cerebellar damage in alcohol-dependence [59], [60], [61], such an alteration does not correlate with episodic memory impairment in alcoholics in the present study.

Our results highlight a double dissociation in memory processes among alcoholics. Indeed, regarding neural substrates, semantic and episodic memory impairment can be dissociated in the alcohol dependent sample studied here. Whereas ADC abnormalities in *cortical* regions underlie semantic memory impairment, ADC abnormalities within *deep* brain regions seem to underlie episodic memory impairment. These results converge with other behavioral results to indicate that episodic memory does not depend on the same memory system that the one mediating semantic memory [62].

Interestingly, a recent study revealed that alcoholics had lower performance levels than controls on the FCSRT but seem to improve their performance at the same rate as them. Thus, alcoholics would appear to require more learning trials to achieve the same results, suggesting a slower pace of learning abilities [2]. Therefore, learning function might be altered but not lost in alcoholics. This might suggest that such a dysfunction is underlined by discrete rather than marked alterations or by damages in specific circuits. Present results demonstrate that DTI-derived measures can be good indicators of structural and functional abnormalities in alcoholism. Grey matter volume decrease, suggestive of neuronal loss, revealed in the same regions as those where an ADC increase was found, seem however unlikely related to episodic memory impairment as no correlation between grey matter volume and episodic memory performance was found in these participants. In fact, the early episodic memory dysfunction in alcoholism may be related to glial cell alteration leading to an insufficient support of neurons; or to axonal damage and demyelination, which can result in an anatomical disconnection of the hippocampus from the main source of its neocortical inputs. Recently, changes of protein expression associated with astrocytes and oxidative stress have been identified in hippocampal regions and may indicate the possibility that increased levels of central nervous system ammonia and reactive oxygen species induced by alcoholic mild hepatic damage/dysfunction could cause selective damage in glial cells of the hippocampus (for review see [63]).

Finally, our results add further evidence against “the bigger is the better” theory, as in alcoholism; the extent and severity of the episodic memory deficits did not seem related to the size of the volume alteration, even though it might be a contributor.

### Limitations

The first limitation in this study is that the use of cross-sectional design cannot lead to causal inferences from our data and thus do not permit any generalization of the data.

Then, since the primary findings of ADC changes are located close to grey matter/liquid interface, the potential contribution from partial volume effects cannot be fully excluded. In the case of partial volume effects, results would reveal higher ADC and also lower FA values in alcoholics. Therefore, a between-group comparison of FA values was carried-out as complementary analyses, and did not reveal any significant results. Furthermore, atrophy in alcoholics does not correlate with an increase in ADC; which further suggests that partial volume effects cannot be the sole cause of the changes observed in ADC values.

Interestingly, neither FA nor ADC changes were found in alcoholics' white matter surrounding hippocampal regions, suggesting that no major axonal projections to or from these regions have degenerated in the alcoholic subjects studied here.

Another limitation stems from tobacco use in the alcoholic population, as most of the alcoholic subjects included in this study were smokers. Neuropsychological performance and brain structural differences were reported between smoker and non-smoker abstinent alcoholics [64]. However, the interaction of smoking and chronic alcoholism is still unclear. Furthermore, statistical analyses between smoker and non-smoker alcoholics found no difference in neuropsychological performance.

Although, the use of only one test of episodic and semantic memory may limit the investigation of brain correlates of episodic memory impairment in alcoholic subjects, the results found in the present study are consistent with previous reports showing that episodic memory performance is associated with specific brain regions [48], [49].

Finally, our findings and interpretations are drawn from a sample of males. Since alcohol-related neuropsychological deficits and brain alterations are gender dependent [65], the questions raised in this study still remain for alcoholic women.

### Conclusion

We report evidence for functionally relevant microstructural changes in uncomplicated alcoholics. These microstructural changes were present in brain regions serving episodic memory functions in healthy subjects (temporal and frontal lobes). These observations support the view that DTI is a useful *in vivo* tool for identifying microstructural cerebral pathology in alcohol dependence and, more specifically to our study, provide preliminary evidence suggesting microstructural alteration as an underlying mechanism for episodic memory impairment in this pathology. Although correlations do not mean causality, exploring the neural correlates of memory performance in alcohol dependents lead us to suggest that specific brain regions play specific roles in the alcoholics' memory functioning. Furthermore, we highlight DTI as a technical support for the exploration of such discrete alterations.

Finally, we suggest that the apparent coefficient of diffusion could be a better correlate of neuropsychological measurements than conventional MRI measures.
